# Improving Pharmacist-Led Pediatric Patient Education on Oral Chemotherapy at Home

**DOI:** 10.3390/children10101656

**Published:** 2023-10-06

**Authors:** Anika Patel, Christopher M. Nguyen, Kristin Willins, Elsabella Y. Wang, Grace Magedman, Sun Yang

**Affiliations:** 1 School of Pharmacy, Chapman University, Irvine, CA 92618, USA; 2 Herbert Wertheim School of Public Health, University of California San Diego, San Diego, CA 92093, USA; 3 CHOC Children’s Hospital, Orange, CA 92868, USA

**Keywords:** oral chemotherapy, patient education, pediatric patients, pharmacists

## Abstract

Oral chemotherapy (OC) has been increasingly used in pediatric patients diagnosed with cancer, which is primarily managed in the outpatient setting. Different from adults, pediatric patients face unique challenges in administering these hazardous medications at home. Because of the complexity of pediatric pharmaceutical care and the hazardous nature of chemotherapy agents, comprehensive patient education is imperative to mitigate the potential safety risks associated with OC administration at home. Pharmacists play a vital role in patient education and medication consultations. However, the lack of practice guidelines and limited resources supporting OC counseling are noted. Additional barriers include insufficient knowledge and training on OC, which can be improved by continuing education. In a regional children’s hospital, a comprehensive OC education checklist was developed for pediatric patients and their caregivers to standardize consultations led by pharmacists. An infographic OC handout was also formulated to improve patient knowledge and awareness. Moreover, innovative approaches such as using telepharmacy, smartphone applications, and artificial intelligence have been increasingly integrated into patient care, which can help optimize OC consultations for children and adolescents. Further studies are warranted to enhance oral chemotherapy education specifically tailored for pediatric patients in outpatient settings.

## 1. Oral Chemotherapy Agents for At-Home Administration

Cancer management has been significantly impacted by innovations in precision medicine in the past two decades. This has led to the increased development and approval of novel oral chemotherapy (OC) agents for targeted therapy. In 2020 alone, the FDA approved ten new oral medications for cancer treatment, with reports estimating that oral medications consist of 25–35% of anti-cancer drugs currently in development [1,2]. OC refers to medications for anti-cancer treatment with different mechanisms of action administered via mouth or enteral feeding tubes [3]. OC drugs include classic oncolytic or cytotoxic drugs (i.e., 6-mercaptopurine, methotrexate, gemcitabine, arsenic trioxide, and temozolomide), drugs for hormonal treatment (i.e., selective estrogen receptor modulators, aromatase inhibitors, GnRH agonists and antagonists, and anti-androgens), and various anti-neoplastic drugs for targeted therapy (i.e., receptor tyrosine inhibitors, and small molecule inhibitors targeting epidermal growth factor receptor, mTOR, KRas, Bcl-2, and fibroblast growth factor receptor) [4,5].

The use of OCs offers many benefits and advantages, including convenience of administration, enhanced patient autonomy and privacy, less disruption in the patient’s daily routine, elimination of the need for parenteral line placement thereby minimizing the risk of infection, and avoidance of transportation and hospitalization. In addition, OCs typically present with lower emetic risk and are associated with milder adverse effects when compared to intravenous (IV) chemotherapeutic drugs, contributing to an improved quality of life for patients while continuing active anti-cancer treatment [6,7]. Drug delivery by this route is far less invasive providing an ease of administration that can allow for treatment in the comfort of the patient’s home. Moreover, many IV chemotherapy agents are vesicants and carry the risk of extravasation, which would be avoided with the use of oral agents. The utilization of OCs has transformed care delivery for oncology patients. Given the convenience and accessibility of OCs, administration of the medication is now predominantly managed by the patients or caregivers, reducing the reliance on healthcare professionals. As a result, there is a significant demand for developing a best practice model for patient education that particularly focuses on the outpatient setting for medication safety.

Cancer is one of the leading causes of death in children (5–14 years) in the United States [8]. OCs demonstrate essential value in pediatric oncology patients. The most common pediatric malignancy is acute lymphoblastic leukemia (ALL), where first-line anti-cancer therapy involves induction-maintenance regimens [9,10]. Once remission is achieved, maintenance chemotherapy using an OC such as 6-mercaptopurine lasts 2 to 3 years to prevent or delay cancer relapse [9,10]. Continuation of anti-cancer therapy at home is of particular benefit for pediatric patients, allowing them to maintain relatively regular routines, reduce their inpatient hospital stays, manage emotional distress associated with hospitalization, and attain an improved quality of life. A study conducted at the Children’s Hospital of Philadelphia found that IV chemotherapy given at home was superior to IV therapy administered in the hospital based on quality-of-life variables including patient’s sense of well-being, appetite, ability to do schoolwork, and improved mood [11]. Parents of the pediatric patients in this study also reported better capacity to keep up with household chores and work responsibilities and spend more time with their spouse and other children in their family.

## 2. Patient Education and Medication Counseling for Children

Pediatric patients who receive medications at home face unique challenges in medication administration and in predicting medication response due to the high percentage of off-label use [12]. There are many notable differences in drug absorption, distribution, metabolism, and excretion observed in children and neonates compared to adults. At different growth stages, the rate of gastric emptying, gastric pH, surface area available for drug absorption, hepatic metabolic enzyme levels, and renal clearance vary significantly [13]. Pediatric patients require individually tailored dosing regimens based on their age and body weight to account for their pharmacokinetic differences. As such, ensuring accurate and age-appropriate doses and regimens is vital for medication safety and therapeutic effectiveness.

In addition, to facilitate oral administration of medications, especially for neonates and infants, liquid formulations are commonly preferred due to the ease of individualized dosing. However, most medications for children are used off-label and may not have age-appropriate formulations to accommodate their needs [12,14]. Extemporaneous compounding of medications may be restricted by lack of information on stability, storage conditions, impact on drug absorption and bioavailability, drug efficacy, and palatability [15,16]. These challenges further complicate the pharmaceutical care provided to children and increase the risk of medication errors due to possible miscalculations, incorrect dilutions and measurements, and the use of inappropriate ingredients [17,18]. Unlike adults who in general can conduct self-care independently, most pediatric patients rely on their parents and other care providers. In a study conducted on outpatient pediatric care, 84% of medical treatment errors were classified as medication errors, with 11% specifically related to medication administration [19]. The primary cause of these reported medical errors was identified as problematic communication and/or insufficient handoffs. Enhancing communication between providers/pharmacists and parents has been shown as an effective approach to mitigating medication errors in pediatric patient care [19].

Medication counseling and education are even more crucial for patients diagnosed with cancer. Oncology patients are at a higher risk of medication errors due to polypharmacy, comorbidities, and the immunocompromising effects of anti-cancer agents [20]. Chemotherapy regimens are frequently intricate and tailored to the individual patient. Studies demonstrated that chemotherapy errors affect 1–3% of adult and pediatric oncology patients, occurring at a rate of about 1–4 per 1000 orders [20]. Medical errors were reported throughout the entire process of anti-cancer treatment, including prescribing, dispensing, administration, and monitoring of chemotherapy agents across diverse healthcare settings. As targeted therapy continues to advance rapidly, oral chemotherapy drugs pose an increasing risk of errors. In an earlier study on OC, 38.8% of errors were reported as incorrect doses and 10% as missed doses [21]. Pediatric patients are more vulnerable to medication errors given the constant dose adjustments based on body weight and the prolonged anti-cancer treatment (e.g., the maintenance therapy for acute lymphoblastic leukemia lasting 2–3 years) [22]. Efforts should be made to address any barriers that hinder effective communication between healthcare providers and patients/caregivers to improve medication counseling and education.

## 3. Essential Subjects for Oral Chemotherapy Counseling in Pediatric Patients

### 3.1. Safety Considerations (Administration, Handling, and Disposal)

Like IV chemotherapeutic medications, OC agents are defined as hazardous drugs given their carcinogenicity, developmental toxicity (including teratogenicity), reproductive toxicity, and genotoxicity [23]. Although OC provides many advantages over IV agents, a shift from infusion to oral administration puts the responsibility of managing these hazardous medications in the hands of the patient and/or their caregivers. The National Institute for Occupational Safety and Health (NIOSH) recommends installing biological safety cabinets and providing personal protective equipment (PPE) to minimize occupational exposure to hazardous medications. United States Pharmacopeia (USP) <800> has also clearly outlined the requirements and regulations on compounding hazardous medications. However, these safety precautions are not applicable in the home setting. Consequently, unintended exposure to caregivers and family members may occur.

OC tablets and capsules are shown to produce particulates that could be released into the air and inhaled. This risk increases particularly when their outer coatings are damaged or if capsules are opened. Exposure may also occur via direct contact with contaminated body fluids or waste, as traces of drug or their active metabolites can be excreted through urine or feces [24]. Depending on their half-life, excretion of the active drug or its metabolites may extend for several days after treatment cessation. The International Society of Oncology Pharmacy Practitioners (ISOPP) recommends that patients on oral 6-mercaptopurine should continue to use PPE when in contact with urine for two days and feces for five days after administration [25]. If caregivers and family members are not aware of the need for stringent safeguards, mishandling in this context can lead to possible direct or indirect hazardous exposure [26].

Extemporaneous compounding of OC medications is becoming common practice when treating pediatric patients who cannot swallow entire tablets or capsules due to their younger age, individualized dosing requirements, or clinical morbidities. Hazardous compounding is generally performed by a well-trained pharmacy technician or pharmacist in a hospital or special compounding pharmacy. However, at home, handling and administration of OC is primarily carried out by patients and their caregivers with limited medical training.

Furthermore, disposal of hazardous medications can be challenging in certain areas, especially in rural or economically disadvantaged communities. Inappropriate disposal of hazardous OC agents can cause environmental contamination and create potential public health concerns. In 2012, Alameda County in California became the first local government to pass legislation requiring pharmaceutical companies to design, fund, and operate a program to safely collect and dispose of unwanted drugs, including hazardous medications [27]. Following that, several other California counties have successfully passed and implemented regulations regarding safe medication disposal [28,29,30,31]. However, an earlier study demonstrated that patients rarely received education and counseling on the proper disposal of unneeded OC drugs [32]. More efforts from all relevant stakeholders including national regulatory agencies and state boards of pharmacy are needed to advocate, develop, and standardize the safe disposal of hazardous medications in the home setting. Utilizing a patient education checklist may also aid pharmacists in ensuring sufficient counseling on the safe disposal of OC.

### 3.2. Medication Adherence for Optimal Treatment Effectiveness

With all the essential information about handling, administering, and disposing of OC, healthcare providers must also emphasize the importance of adherence in maximizing the benefits of their treatment. Inadequate adherence to chemotherapy regimens results in a shorter duration of remission, lack of complete response to the medication, increased costs of medical treatment, and overall reduced quality of life [33]. Many factors contribute to medication nonadherence in pediatric patients, including lack of understanding about the disease or treatment, socioeconomic status, drug taste, and the medication administration schedule [34]. In addition, certain psychosocial and demographic issues in oncology patients also adversely impact their medication compliance, such as comorbid conditions, depression, unmanaged side effects, and limited access to medications due to cost or location [35,36].

Adolescents (aged 12–18 years old) are known to be a particularly nonadherent group [37], and studies showed that 21–60% of adolescents and young adults with cancer are nonadherent to oral medications [38]. Although many factors contribute to their struggle with medication compliance, dosing medications during school time is a notable obstacle [34]. Potential solutions include utilizing technology to help remind them when to take their medications, altering the dosing schedule to take the next dose after school, and educating these patients while being cognizant of social stigma [34]. Supplementing patient education with motivational interviewing techniques has proven to be efficacious and feasible in improving OC compliance [39]. Healthcare providers should address potential barriers to compliance and explore solutions to ensure patients are adherent to their medication regimens and derive the maximum benefit from their treatment.

Caregivers are essential in providing and supporting pediatric medical care at home and ensuring medication adherence, especially for younger children [40,41]. A study showed that pediatric patient care provided by caregivers can be influenced by the attitude of the child toward the treatment [42]. As anti-cancer medications are always riddled with adverse reactions and side effects, child resistance to medication administration is very common and can be a real challenge for caregivers [42]. In an earlier study, 65% of caregivers of pediatric patients who were discharged home were given instructions on medication dosing, but none of them were taught how to administer the medication at home [43]. In addition, multigenerational care is common in pediatric patients and is often conducted by multiple caregivers [44]. Factors such as aging, low health literacy, and language barriers may restrict their ability to provide optimal medical care. It is crucial to equip all caregivers with the skills and resources to address these potential challenges in OC administration at home [45,46].

## 4. Optimizing Pharmacist-Led Education on OC in Pediatric Patients

OC allows for the continuation of appropriate anti-cancer treatment delivered primarily by patients and their caregivers at home. Essential for effective OC therapy is the provision of comprehensive patient education to ensure both safe medication use and adherence. Research has shown that patients consistently rank pharmacists among the top trusted healthcare professionals [47]. A systematic review involving adult outpatients with cancer revealed that pharmacist interventions significantly decreased cancer-related adverse events and medication side effects while increasing medication adherence and patient quality of life [48]. As such, studying, developing, and implementing innovative strategies to ensure sufficient patient education and counseling by pharmacists is essential to improving patient care with OC.

Several healthcare institutions have initiated pharmacist-led oral oncology programs to provide comprehensive pharmaceutical care and patient education support [49,50]. In collaboration with the patient’s healthcare team, pharmacists provide a broad range of expertise to assist in many aspects of patient care including ensuring safe compounding and dispensing of chemotherapy agents, reducing drug waste, minimizing inappropriate exposure to hazardous drugs, developing institutional policy and guidelines, assisting in making evidence-based therapy decisions, providing supportive care, and contributing to cancer research through assistance in clinical trials and supporting investigational drug service programs [51]. In addition, pharmacists can help develop educational materials and programs regarding potential drug interactions, adverse effects of medications, and symptom management. Such programs have resulted in improvements in patient understanding and satisfaction with their OC treatment. The role of pharmacists in OC clinics is not only limited to clinical monitoring and patient education but also includes reaching out to patient insurance companies to ensure their chemotherapy medications will be covered. In addition, pharmacists are well equipped with the knowledge and skills to determine the appropriate administration of OC when patients cannot swallow whole capsules or tablets, commonly seen in younger children and during active anti-cancer treatment due to toxicities.

Given the complexity of anti-cancer therapy, in general, a multidisciplinary team including physicians, nurses, and pharmacists works collaboratively to carry out treatment and provide patient education. Despite the setting of clinical practice, clear communication of expectations regarding patient education and care delegation is vital. Every team member should understand their role and responsibilities, including what needs to be taught to the patient, who will do it, and when. More importantly, every patient care team member should stay updated with ongoing training and education to ensure they have the knowledge to perform their roles effectively in patient education, particularly on OC. Another essential aspect is to ensure appropriate documentation of all patient education and communications. This helps in tracking and monitoring the patient’s treatment progress and ensuring continuity of care.

### 4.1. Challenges and Barriers to Providing Patient Education on OC

Although the safe handling of hazardous drugs is deemed imperative across the healthcare continuum, few guidelines delineate specific recommendations for safe handling and disposal when utilized in the home setting [52]. Additional barriers affecting adequate patient education on OC include circulating misconceptions of safety and an overall insufficiency of knowledge. Particularly, OC dosage forms may be perceived as less toxic with minimal side effects in comparison to IV therapy due to the ease of administration [53,54]. An earlier survey involving community pharmacists found that they were most proficient in general dosing but less knowledgeable about adverse effects, safe handling, and disposal [54]. About 95% of pharmacists indicated their pharmacies did not use separate counting trays for hazardous drugs [54]. Notable insufficiencies of knowledge on OC safe handling and disposal were also identified in a recent study, where 62.5% of pharmacists believed that OC is safer than IV chemotherapy. The greatest barrier to OC counseling was insufficient training [53]. As a consequence, the overall level of awareness in patients remains less than optimal [27,28,29,30,31,53].

The role of community pharmacists in providing patient care has expanded over the years to now include administering vaccines, participating in point-of-care testing, and conducting medication therapy management. As their responsibilities have increased, less time may be allocated for patient consultations. Customer service is highly regarded in the community, and long pharmacy wait times could jeopardize optimal patient care. An earlier study found that 75% of community pharmacists reported burnout due to emotional exhaustion, depersonalization, and reduced personal accomplishment, particularly in chain pharmacies [55,56]. This may be associated with the pressure to meet certain performance metrics based on prescription volume instead of value. Consistently, community pharmacists are primarily reimbursed for dispensing medications rather than for providing effective pharmacy services and addressing the clinical needs of patients, which restricts them from spending a suitable amount of time on patient counseling. Furthermore, given the complexity of pediatric pharmacotherapy, pediatric patients and caregivers may need more intensive consultation on their medications. This requires additional time to ensure adequate understanding.

### 4.2. Possible Solutions to Improve OC Education in Pediatric Patients

#### 4.2.1. Bridging the Knowledge Gap on OC

The Occupational Safety and Health Administration (OSHA) requires all pharmacy personnel to receive training on the safe handling of hazardous drugs. Such practices are more heavily enforced in inpatient settings and specialty pharmacies, where handling, compounding, and dispensing hazardous drugs occur regularly. In community pharmacies, however, only a very small fraction of commonly dispensed medications are hazardous; therefore, the pharmacist’s experience and training on OC may be limited [57]. To bridge the knowledge gap, mandated self-study or training on handling hazardous medications and related regulatory updates should be incorporated into pharmacist continuing education and assessed on a regular basis. Professional organizations may offer specific workshops or training sessions that can provide complimentary training to pharmacists, especially those working in smaller institutions or independent pharmacies with limited resources. Pharmacists can also refer to diverse resources (e.g., professional organizations, FDA, board of pharmacy) to obtain information, such as recipes for extemporaneous compounding of OC oral solutions on the HOPA website [58].

#### 4.2.2. Developing a Comprehensive Patient Counseling Checklist

**Figure 1:**
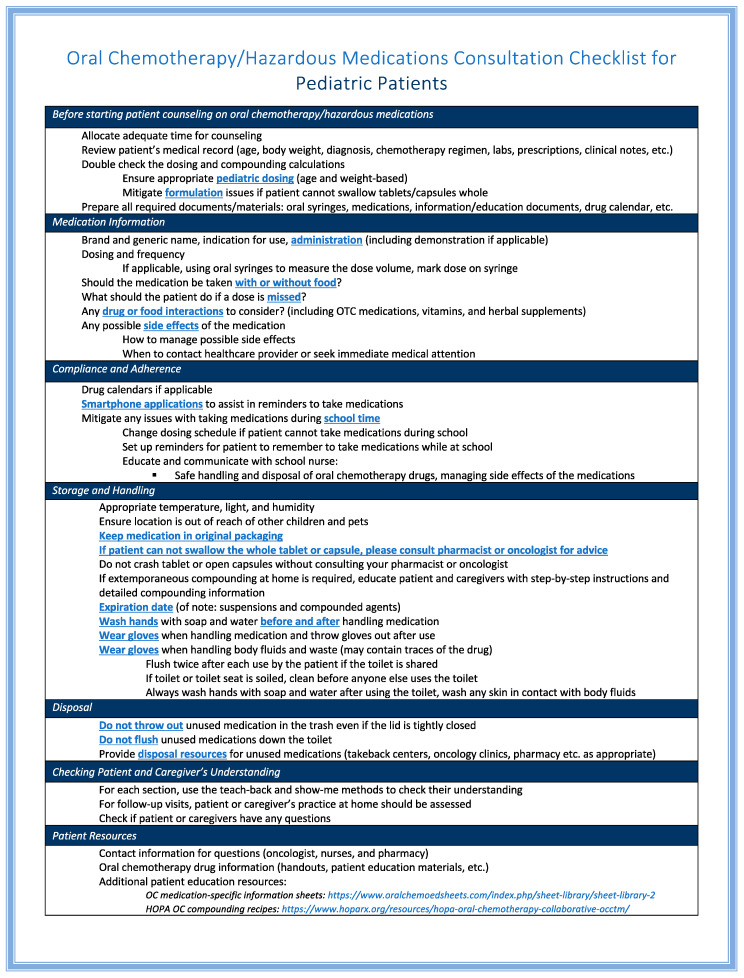
Oral chemotherapy consultation checklist to assist pharmacists in providing comprehensive medication information to pediatric patients and their caregivers. “https://www.oralchemoedsheets.com/index.php/sheet-library/sheet-library-2 (accessed on 4 August 2023)”. “https://www.hoparx.org/resources/hopa-oral-chemotherapy-collaborative-occtm/ (accessed on 4 August 2023)”.

An Oral Chemotherapy/Hazardous Medications Consultation Checklist for Pediatric Patients (Figure 1) has been developed at a regional children’s hospital based on literature, guidelines, professional standards, and patient education materials [3,52,53,58,59,60,61]. This checklist aims to help pharmacists prepare and standardize patient consultations across different settings, and ensure all critical information is explained and discussed with the patient and caregivers. Upon OC dispensing, the chemotherapy regimen should be available for pharmacists to review and double-check the indication, dose, and frequency, which provides additional safeguards for patient safety.

#### 4.2.3. Providing Informational Handouts with Oral Chemotherapy Prescriptions

In the age of telemedicine, medications may often be delivered to patients’ homes directly and consultations should be provided in an innovative manner to ensure vital information is successfully delivered and well received. To minimize barriers to patient education and access to health information resources, pharmacists can provide OC information sheets with the medications to ensure an accurate and adequate understanding of the proper administration, handling, storage, and disposal of these agents at home. For people who are not proficient in the use of technology, particularly computers, software, and digital devices, using traditional written/verbal instructions remains the primary approach for patient education. Studies showed that patients prefer a combination of verbal and written information that they can refer to, such as patient handouts and medication calendars [62]. Patient education handouts are commonly used in OC investigational drug trials to ensure that drug safety is appropriately addressed [63]. At our regional children’s hospital, in addition to standardized patient counseling, we use an infographic information sheet to emphasize the safe handling and disposal of OC agents at home. Ideally, this information should be distributed to all members involved in pediatric patient care including family members, other caregivers, and even school nurses who may be involved in the administration of OC to the child.

**Figure 2:**
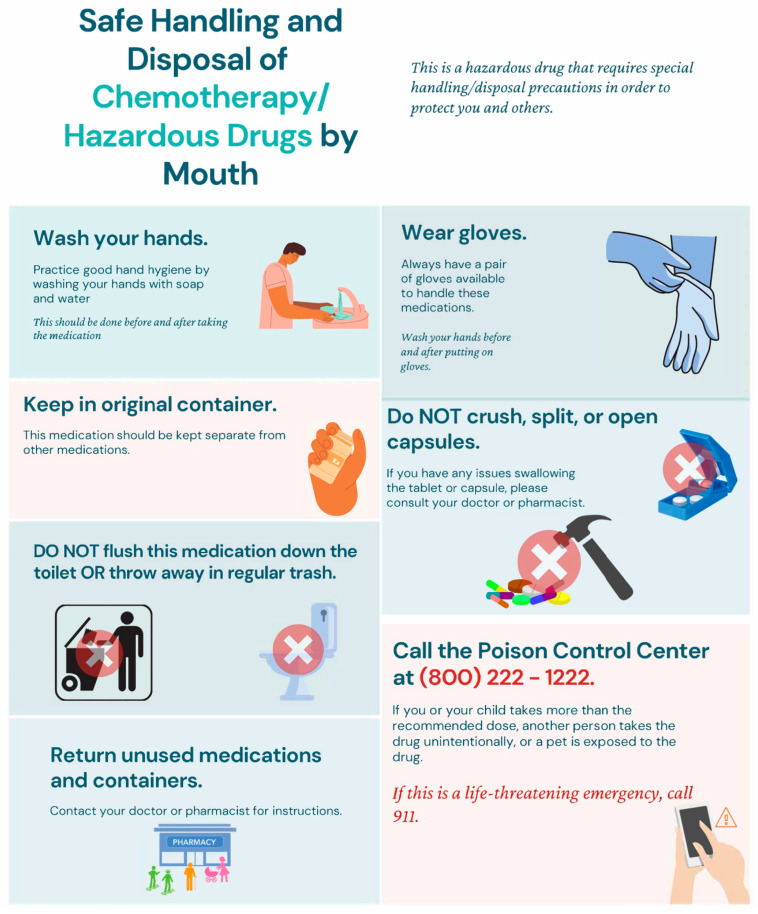
A chemotherapy/hazardous drug infographic handout developed for pediatric patients and their caregivers.

The American Society of Clinical Oncology (ASCO) and the Oncology Nursing Society (ONS) guideline emphasizes that these educational materials are made with the patient’s interests in mind and that the information presented is comprehensible [59]. An infographic patient handout, specifically curated for pediatric patients, was created following the ASCO/ONS guideline (Figure 2). It highlights the areas requiring improvement in patient education and addresses the knowledge gaps identified in a recent survey study [53]. Various elements were taken into account to ensure the information presented can be easily understood, recalled by memory, and retained credibility. The handout was limited to one page to not overwhelm the patient with information and to ensure that it could be efficiently reviewed with the provider and act as a resource for future reference. It was prepared in a stepwise approach to guide the patient/caregiver as they prepare to administer the OC. The written material was supplemented with visuals which were carefully chosen to allow patients to instantly recall important details about their medications. The use of visuals could also help overcome potential language barriers. In addition, the handout contains a QR code to refer patients and caregivers to credible public resources for additional instructions on managing side effects, medication handling, storage, and disposal. A future study could evaluate the effectiveness of the handout to ensure it enhances the patient’s learning experience.

#### 4.2.4. Developing Smartphone or Online Applications to Assist in Patient Education

Future endeavors to improve patient education could include the development of a pediatric-friendly smartphone application to help present information on OC agents. It should be highly interactive to engage younger patients. Complex information should be simplified and represented in a more child-receptive manner to provide the best care and understanding. Adolescents and young adults are well adapted to technology and have a higher willingness to learn via Information and Communications Technology devices, in which case traditional paper-based education materials are potentially less effective [64]. Additionally, technology can help bridge barriers in patient education for those who are vision-impaired, hearing-impaired, or require language translations.

An application for OC agents can be a powerful tool to assist in patient education. It provides the patient with direct access to comprehensive information and a direct line of communication between the patient and provider for questions or concerns [65]. The patient portal within the application can also allow the healthcare provider to track medication usage on their end and provide more comprehensive and timely care. Medication adherence, refill alerts, and drug interaction information could be integrated into the application to promote effective and safe treatment [65]. Furthermore, a search feature can provide drug take-back locations near the patient’s residence to facilitate proper OC disposal.

#### 4.2.5. Maximizing Telepharmacy Potential for Patient Education

Telemedicine has become increasingly integrated into patient care, which has shown many benefits in improving diagnostic accuracy, reducing costs, and decreasing mortality rates, especially during the pandemic [66]. In the U.S., 23 states explicitly allow telepharmacy practice, while 11 additional states have laws and regulations in place that either permit waivers or have initiated pilot pharmacy practice programs [67]. The most observed telepharmacy initiatives during the COVID-19 pandemic were virtual consultations, home delivery of medicines, and patient education [68]. The implementation of telepharmacy enhances direct interactions between patients and healthcare providers, enabling patients to access timely pharmaceutical services while ensuring the safe handling and administration of medications in their own homes. Furthermore, alongside immediate teach-back, integrating follow-up phone calls may further enhance patient education and monitoring. While holding promise, using virtual platforms for patient counseling and education still faces several practical obstacles that need to be overcome, such as technology, financial reimbursement, language, and licensure [69,70,71]. Further investigations on risk-benefit assessment and the impact of telepharmacy on patient safety and outcomes are warranted. Appropriate legislation and reimbursement, ensuring insurance coverage are essential to maximize the benefits of telepharmacy for patient education.

#### 4.2.6. Utilizing Artificial Intelligence to Optimize Patient Education

The development of artificial intelligence (AI) technology has empowered clinicians, including pharmacists, to provide patient care in a myriad of innovative ways and transformed how healthcare is delivered [72,73]. By analyzing vast accumulated data, AI technology may help uncover patterns and predict the patient’s medication compliance based on previous medication refill records and specific demographic aspects such as age, education, and socioeconomic status [73]. This information generated by AI can help pharmacists tailor their counseling to improve the quality and efficacy of patient education and to ensure patient-centric care.

Furthermore, the integration of AI and automation systems can assist in improving the overall functional capacity of pharmacies by optimizing the pharmacy staff’s time and increasing the efficacy of operations [74,75]. As a result, pharmacists will be able to focus on patient-care tasks and perform medication counseling, reassess patient understanding, demonstrate drug administration techniques, proactively track and monitor medication adherence, and overall provide exceptional pharmacy care to patients.

## 5. Conclusions

The utilization of OC agents at home has increased in the pediatric population in recent years. Pharmacists need to ensure adequate patient consultations are provided to optimize medication safety [38]. Pediatric patients and caregivers should be educated on the safe and effective use of their OC medications at home. Strategies discussed in this review may help healthcare providers, including pharmacists, improve patient education on OC. Developing a best practice model incorporating multiple approaches is warranted to enhance OC treatment in outpatient settings.
